# Reductive Power Generated by *Mycobacterium leprae* Through Cholesterol Oxidation Contributes to Lipid and ATP Synthesis

**DOI:** 10.3389/fcimb.2021.709972

**Published:** 2021-07-28

**Authors:** Thabatta L. S. A. Rosa, Maria Angela M. Marques, Zachary DeBoard, Kelly Hutchins, Carlos Adriano A. Silva, Christine R. Montague, Tianao Yuan, Julio J. Amaral, Georgia C. Atella, Patrícia S. Rosa, Katherine A. Mattos, Brian C. VanderVen, Ramanuj Lahiri, Nicole S. Sampson, Patrick J. Brennan, John T. Belisle, Maria Cristina V. Pessolani, Marcia Berrêdo-Pinho

**Affiliations:** ^1^ Laboratório de Microbiologia Celular, Instituto Oswaldo Cruz, Fundação Oswaldo Cruz, Rio de Janeiro, Brazil; ^2^ Department of Microbiology, Immunology and Pathology, Colorado State University, Fort Collins, CO, United States; ^3^ Department of Microbiology and Immunology, Cornell University, Ithaca, NY, United States; ^4^ Department of Chemistry, Stony Brook University, Stony Brook, NY, United States; ^5^ Laboratório de Química Biológica, Diretoria de Metrologia Aplicada às Ciências da Vida, Instituto Nacional de Metrologia, Qualidade e Tecnologia, Rio de Janeiro, Brazil; ^6^ Laboratório de Bioquímica de Lipídeos e Lipoproteínas, Instituto de Bioquímica Médica, Universidade Federal do Rio de Janeiro, Rio de Janeiro, Brazil; ^7^ Divisão de Pesquisa e Ensino, Instituto Lauro de Souza Lima, Bauru, Brazil; ^8^ Departmento de Controle de Qualidade, Instituto de Tecnologia em Imunobiológicos, Fundação Oswaldo Cruz, Rio de Janeiro, Brazil; ^9^ Department of Health and Human Services, Health Resources and Services Administration, Healthcare Systems Bureau, National Hansen’s Disease Programs, Baton Rouge, LA, United States

**Keywords:** *Mycobacterium leprae*, cholesterol, cholestenone, PGL-I, PDIM, 3β-HSD, reductive power, oxidation

## Abstract

Upon infection, *Mycobacterium leprae*, an obligate intracellular bacillus, induces accumulation of cholesterol-enriched lipid droplets (LDs) in Schwann cells (SCs). LDs are promptly recruited to *M. leprae*-containing phagosomes, and inhibition of this process decreases bacterial survival, suggesting that LD recruitment constitutes a mechanism by which host-derived lipids are delivered to intracellular *M. leprae*. We previously demonstrated that *M. leprae* has preserved only the capacity to oxidize cholesterol to cholestenone, the first step of the normal cholesterol catabolic pathway. In this study we investigated the biochemical relevance of cholesterol oxidation on bacterial pathogenesis in SCs. Firstly, we showed that *M. leprae* increases the uptake of LDL-cholesterol by infected SCs. Moreover, fluorescence microscopy analysis revealed a close association between *M. leprae* and the internalized LDL-cholesterol within the host cell. By using *Mycobacterium smegmatis* mutant strains complemented with *M. leprae* genes, we demonstrated that *ml1942* coding for 3β-hydroxysteroid dehydrogenase (3β-HSD), but not *ml0389* originally annotated as cholesterol oxidase (ChoD), was responsible for the cholesterol oxidation activity detected in *M. leprae*. The 3β-HSD activity generates the electron donors NADH and NADPH that, respectively, fuel the *M. leprae* respiratory chain and provide reductive power for the biosynthesis of the dominant bacterial cell wall lipids phthiocerol dimycocerosate (PDIM) and phenolic glycolipid (PGL)-I. Inhibition of *M. leprae* 3β-HSD activity with the 17β-[N-(2,5-di-t-butylphenyl)carbamoyl]-6-azaandrost-4-en-3one (compound 1), decreased bacterial intracellular survival in SCs. In conclusion, our findings confirm the accumulation of cholesterol in infected SCs and its potential delivery to the intracellular bacterium. Furthermore, we provide strong evidence that cholesterol oxidation is an essential catabolic pathway for *M. leprae* pathogenicity and point to 3β-HSD as a prime drug target that may be used in combination with current multidrug regimens to shorten leprosy treatment and ameliorate nerve damage.

## Introduction

Leprosy is a chronic infectious disease caused by *Mycobacterium leprae*, an obligate intracellular bacillus preferentially found in dermal macrophages and Schwann cells (SC) of peripheral nerves. Although effective treatment for leprosy has been a reality for decades, neural damage remains a common outcome of the disease, and is often irreversible (Lockwood and Saunderson, 2012). Despite the sterling ongoing efforts from the World Health Organization (WHO) to eliminate the disease using a 3-drug global control strategy, leprosy remains endemic in many regions of the world with almost 250,000 new cases of leprosy reported yearly. According to the WHO, Brazil represented 93% of all leprosy incidence in the Americas, and together with India and Indonesia, account for 79.6% of all the new cases detected globally (WHO, 2019). Although leprosy is a very ancient disease, an understanding of its pathogenicity is still limited, being hampered by the absence of experimental models and the failure to cultivate the pathogen *in vitro*. The inability to culture this bacterium *in vitro* is associated with dramatic reduction of genetic capability of *M. leprae*. *M. leprae* underwent reductive evolution throughout the eons loosing one-half of its potential coding capacity, compared to other mycobacteria and conserving what is considered the minimal set of genes of a pathogenic *Mycobacterium* spp. (Cole et al., 2001).

Host lipids play an important role in *M. leprae* metabolism and mycobacterial infections in general (Kim et al., 2010). *M. leprae* stimulates lipid droplet (LD) accumulation in the host cell (Tanigawa et al., 2008; Mattos et al., 2010; Mattos et al., 2011a; Mattos et al., 2011b), generating the host cell foamy phenotype, which has been a hallmark of leprosy lesions since Virchow (Virchow, 1863). The host lipid droplets in foamy cells are rich in neutral lipids, such as triacylglycerols, cholesterol, and cholesterol esters and, indeed, cholesterol is one of the main lipids enriched in foamy cells during *M. leprae* infection (Mattos et al., 2010; Mattos et al., 2011a; Mattos et al., 2014). In fact, *M. leprae* infection in macrophages increases the expression of key enzymes in the cholesterol biosynthetic pathway, such as 3-hydroxy-3-methyl-glutaryl-CoA reductase (HMGCR), as well as an enhanced uptake low-density lipoprotein (LDL) receptor (Mattos et al., 2014). Moreover, LDs are promptly recruited to *M. leprae*-containing phagosomes in infected SCs. Inhibition of this process decreases bacterial survival, suggesting that LD recruitment constitutes a mechanism by which host-derived lipids are delivered to intracellular *M. leprae* (Mattos et al., 2011a). Another potential source of cholesterol for *M. leprae* uptake in nerve cells was recently described. The leprosy bacillus accelerates myelin breakdown, a cholesterol-rich membrane. An inhibition of this phenomenon affected bacterial survival (Mietto et al., 2020). Additionally, inhibition of the host cell cholesterol biosynthesis through treatment with statins markedly decreased *M. leprae* survival in macrophages (Mattos et al., 2014) and reduced its survival in the Shepard mouse model of leprosy (Lobato et al., 2014), further suggesting a relevant role for cholesterol during *in vivo* growth of *M. leprae*.

Mycobacteria in general, including *Mycobacterium tuberculosis*, catabolize cholesterol as a carbon and energy source (van der Geize et al., 2007; Pandey and Sassetti, 2008; Thomas et al., 2011a; Uhía et al., 2011). This is not the case for *M. leprae*, as genome sequencing predicted that all genes involved in cholesterol catabolism are non-functional pseudogenes with the one exception: the enzyme responsible for the oxidation of cholesterol to cholest-4-en-3-one (cholestenone), the first step of sterol ring degradation (Cole et al., 2001). Indeed, by using ^14^C-labeled cholesterol derivatives, it was elegantly demonstrated that the cholesterol carbon atoms from both the sterol framework and the aliphatic side chain are not used by *M. leprae* for lipid synthesis and CO_2_ generation (Marques et al., 2015). However, *M. leprae* avidly incorporates cholesterol, converting it to cholestenone both *in vitro* and *in vivo* (Marques et al., 2015). In the same study, 3β-hydroxysteroid dehydrogenase (3β-HSD; encoded by *hsd*, *ml1942)* was regarded as the most likely candidate responsible for this step (Marques et al., 2015) based on its high homology with *M. tuberculosis* 3β-HSD, which displays cholesterol oxidizing activity (Yang et al., 2007; Yang et al., 2011). A second enzyme, cholesterol oxidase (gene is named *choD*) is annotated as a putative cholesterol oxidase in several mycobacterial genomes based on homology with the well-characterized cholesterol oxidases from *Streptomyces* and *Rhodococcus* (Navas et al., 2001; Brzostek et al., 2007). Since *choD (ml0389)* is also present in the ML genome, this enzyme remained as a possible source of cholesterol oxidation.

3β-HSD represents an extensive family of enzymes that oxidizes steroid substrates using pyridine nucleotides (NAD^+^) or their phosphorylated forms (NADP^+^) as electron acceptors (Lachance et al., 1990). In mammals this enzyme is implicated in the generation of steroid hormones, and utilizes as substrates pregnenolone and androstenedione, among others (Simard et al., 2005). The cofactors and their reduced forms, NADH and NADPH, are essential for basic catabolic and anabolic metabolism, respectively. NADPH is a crucial reductant used in lipid anabolism including synthesis of important components of the mycobacterial cell wall (Minnikin et al., 2002). The 3β-HSD of *M. tuberculosis* is described as using cholesterol as the substrate and NAD^+^ as the main enzyme cofactor, and could be inhibited by trilostane [(2R,4R,5R,17α)-4,5-epoxy-17-hydroxy-3-oxoandrostane-2-carbonitrile] (Yang et al., 2007), an androstenediol that is often used as an inhibitor of mammalian 3β-HSD (Potts et al., 1978). More recently, new compounds from a different class of steroids, known as 6-azaandrost-4-en-3-ones (Frye et al., 1993), were generated as specific inhibitors of *M. tuberculosis* 3β-HSD. Some analogs have been shown to effectively inhibit *M. tuberculosis* 3β-HSD, including 17β-[N-(2,5-di-t-butylphenyl)carbamoyl]-6-azaandrost-4-en-3one (compound 1), which has emerged as a powerful tool for better characterization of mycobacterial 3β-HSD (Thomas et al., 2011b). The capacity of compound 1 to inhibit the enzymatic activity of the recombinant *M. tuberculosis* 3β-HSD (IC50 of 23 µM) was demonstrated (Thomas et al., 2011b).

Accordingly, in this present work, we investigated the biochemical relevance of cholesterol oxidation in *M. leprae*-SC interaction. Firstly, we confirmed that 3β-HSD, not ChoD, is the enzyme responsible for cholesterol oxidation in *M. leprae*. Of note, we showed that 3β-HSD activity generates electron donors that may be used by *M. leprae* for ATP synthesis and for the biosynthesis of key cell wall lipids, and that *M. leprae* 3β-HSD inhibition with the compound 1 decreased bacterial intracellular survival in SCs. We conclude from this study that oxidation of cholesterol to cholestenone is a crucial metabolic activity of *M. leprae* and, in contributing to its intracellular survival in SCs, is implicated in nerve colonization and subsequent damage.

## Material and Methods

### Mycobacterial Strains and Human Schwann Cell Culture

*M. leprae*, the Thai-53 strain, was obtained from the hind footpad of athymic *nu/nu* mice, generated at Instituto Lauro de Souza Lima, Bauru, SP, Brazil, and isolated as previously described (Trombone et al., 2014) or prepared at the National Hansen’s Disease Program, Laboratory Research Branch, Louisiana State University, Baton Rouge, LA. Axenic media for *M. tuberculosis* strain mc^2^6230 culture and for *M. leprae* experiments were previously described (Marques et al., 2015). *M. smegmatis* strain mc^2^155 and mutant strains were grown at 37°C in an orbital shaker at 250 r.p.m. in 457 minimal salt medium (Uhía et al., 2011) containing 0.05% tyloxapol (457 MSM-Ty) and complemented mutants were grown on LB broth plus 0.05% tyloxapol (LB-Ty) with 100 μg/ml hygromycin and 25 μg/mL kanamycin as required.

Human SC from ST88-14 tumor cell line originated in a malignant schwannoma, isolated from type 1 neurofibromatosis patients, was kindly donated by Dr. J. A. Fletcher (Dana Farber Cancer Institute, Boston, MA). Cells were cultured in RPMI 1640 media (ThermoFisher Scientific, Waltham, MA) supplemented with 10% fetal calf serum (FCS) (Cripion Biotecnologia, Andradina, Brazil) and maintained at 37°C in 5% CO_2_ atmosphere.

### Genetic Manipulation of *M. smegmatis*

All genetic deletions were generated in *M. smegmatis* mc^2^155. The *Δmsmeg_1604* mutant strain was engineered using mycobacterial recombineering as described (Van Kessel and Hatfull, 2008). Briefly, bacteria containing the recombineering plasmid (pJV53) were induced with 0.2% acetamide and electroporated with a linear recombineering substrate to insert a lox-hygromycin-lox cassette into the *msmeg_1604* ORF at 731 bp. The lox-flanked hygromycin resistance cassette was excised by expressing the Cre recombinase from the plasmid (pTL7bv). The *Δmsmeg_5228* mutant strain was created using the mycobacterial ORBIT method as described (Murphy et al., 2018). Briefly, bacteria containing the ORBIT plasmid (pKM444) were induced with 500 ng/mL anhydrotetracycline and electroporated with a payload vector (pTL11-zeo) and a targeting oligonucleotide to replace the entire *msmeg_5228* ORF sequence (1–1071 bp) with a lox-zeocin-lox cassette. The lox-flanked zeocin resistance cassette was excised by expressing the Cre recombinase from the plasmid (pTL7bv). The *Δmsmeg_1604/Δmsmeg_5228* double mutant was generated by recombineering. The unmarked *Δmsmeg_5228* mutant containing the plasmid (pJV53) was induced with 0.2% acetamide and electroporated with a linear substrate to insert a lox-hygromycin-lox cassette into the *msmeg_1604* ORF at 731 bp. All engineered mutations were verified with Sanger sequencing.

The *M. leprae* gene *ml1942* was synthesized by GenScript (Piscataway, NJ) generating an optimized sequence containing *Nde*I and *Hin*dIII restriction sites at 5’ and 3’ ends, respectively. The *ml1942* construct was ligated into the expression vector pST-KT (Parikh et al., 2013) digested with the same enzymes generating the recombinant plasmid pMRLB121. All Strains and plasmids used in this study are presented in **Table 1**.

**Table 1:** Bacterial strains and plasmids used in this study.

Strain or plasmid	Selectable phenotype	Genotype and/or description	Source or reference
***Mycobacterium smegmatis***			
	None	*M. smegmatis* mc^2^155 △*msmeg_1604*	This study
	None	*M. smegmatis* mc^2^155 △*msmeg_5228*	This study
	Hyg^R^	*M. smegmatis* mc^2^155 △*msmeg_1604/*△ *msmeg_5228*	This study
	Hyg^R^, Kan^R^	*M. smegmatis* mc^2^155 △*msmeg_1604/*△ *msmeg_5228* complemented with pMRLB121	This study
	Hyg^R^, Kan^R^	*M. smegmatis* mc^2^155 △*msmeg_1604/*△*msmeg_5228* complemented with pMRLB122	This study
***Escherichia coli***			
Top 10		F-*mcrA* Δ(*mrr-hsd*RMS-*mcr*BC) Φ80*lac*ZΔM15 Δ*lac*X74 *rec*A1 *ara*D139 Δ(*araleu*)7697 *gal*U *gal*K *rps*L (StrR) *end*A1 *nup*G	Invitrogen
**Plasmid**			
pGEM^®^-T Easy Vector	Amp^R^	Cloning vector	Promega
pST-KT	Kan^R^	*E. coli*-mycobacteria shuttle vector Expression vector, UV15 promoter	Addgene
pMRLB121	Kan^R^	pST-KT:*ml1942*	This study
pMRLB122	Kan^R^	pST-KT:*ml0389*	This study

The *M. leprae* gene *ml0389* (*choD*) was PCR amplified from *M. leprae* genomic DNA with Q5 DNA polymerase (New England Biolabs Inc., Beverly, MA). PCR amplification was performed with the forward primer 5’-gcatatgaagccggattatgacgtcttaatcatc and 3’cccaagcttctatagccaccgcagcgctcc designed to introduce *Nde*I and *Hin*dIII sites to the 5’ and 3’ ends, respectively. The *ml0389* PCR product was ligated into pGEM^®^-T Easy Vector (Promega, Madison, WI) and transformed into competent *Escherichia coli* TOP10 cells. The *ml0389* gene was subcloned into the expression vector pST-KT resulting in the recombinant plasmid pMRLB122. The sequences of the cloned *ml0389* and *ml1942* genes were confirmed by automated nucleotide sequencing. Freshly made competent *M. smegmatis* cells (200 μL) were electroporated with 200 to 400 ng of plasmid DNA. Transformed cells were plated on LB agar containing 100 μg/ml hygromycin and 25 μg/mL kanamycin and incubated at 37°C for 3 days.

### Metabolic Labeling and Assays

To assess recombinant 3β-HSD activity, *M. smegmatis* strains were cultured in 10 mL of LB-Ty broth or 457 MSM-Ty. *M. smegmatis* mutant strain Δ*msmeg_1604*/Δ*msmeg_5228* complemented with pMRLB121 or pMRLB122 was grown to an optical density at 600 nm of 0.6, induced with 50 ng/mL of anhydrotetracycline (ATc), and further incubated for approximately 14 h. Cells were washed and suspended with 200 μL 457 MSM-Ty minus glycerol containing 1 μCi/mL of [4-^14^C]cholesterol (American Radiolabeled Chemicals, Inc., Saint Louis, MO), and incubated at 37°C for 30 min or 2h. Radiolabeled bacteria were separated from spent medium and washed as described previously (Marques et al., 2015). Radiolabeled compounds were resolved by thin-layer chromatography (TLC) using silica gel G60 TLC plates (Millipore, Temecula, CA) developed in petroleum ether-ethyl acetate (1:1) and detected using a Phosphor Imager Typhoon 9400 scanner (GE Healthcare, Sunnyvale, CA). Gene deletion and *M. leprae* genes complementation were assessed by qRT-PCR. Briefly, RNA was extracted using TRizol reagent (Thermo) as recommended by the manufacturer after breaking the mycobacteria in tubes containing 1.0 µm silica microspheres in three cycles of 4000 rpm/45s in the bead-beater. cDNA was synthesized using GoScript random mix (Promega) and the qRT-PCR reaction was performed using primers at the concentration of 400nM (**Table 2**).

**Table 2:** *M. leprae* and *M. smegmatis* qRT-PCR primer sequences.

Primer	Sequence
ML1942 Fw	5’- CGGGAGTAAGAACGCCAAAC-3’
ML1942 Rv	5’- ACCACACGCCTTGATGATTG-3’
ML0389 Fw	5’-GGGAGGCGGTTCGTTGAA-3’
ML0389 Rv	5’-CGGTGAAGGTCGGGTTACAA-3’
MSMEG_5228 Fw	5’- GACGAAACCCTGCCGTACA-3’
MSMEG_5228 Rv	5’-GAACACCTTGCGGAACATCG-3’
MSMEG_1604 Fw	5’- AGAACACGCTGCTCAAGAACTA-3’
MSMEG_1604 Rv	5’- CTTGTCCTTACGTGCCCACC-3’
MSMEG_3757 (16S) Fw	5’- GGGAGCGAACAGGATTAGATAC-3’
MSMEG_3757 (16S) Rv	5’- CCTTTGAGTTTTAGCCTTGCG-3’
SigA Fw	5’-GCCGAGAAGGGCGAGAAG-3’
SigA Rv	5’-GGTTCGCCTCCAGCAGATG-3’

To evaluate cholesterol and palmitic acid utilization and β-oxidation by *M. leprae*, live *M. leprae* (1.6 x10^8^ bacilli) was incubated at 33°C for 1 h with compound 1 with agitation in axenic medium. [26-^14^C]cholesterol (1 µCi/mL) (Quotient Bioresearch Ltd., Cardiff, UK) and/or [1-^14^C]palmitic acid (1 µCi/mL) (American Radiolabeled Chemicals, Inc.) were added to the cultures and the bacilli incubated at 33°C for 48 h in the presence of a filter paper strip saturated with 2 N NaOH for radiorespirometry assay (Buddemeyer, 1974; Marques et al., 2015). Bacilli were collected by centrifugation, lipids were extracted as described (Marques et al., 2015) and 10,000 d.p.m. from lipids extracts were resolved by TLC for cholestenone separation as previously described (Marques et al., 2015). A mobile phase of chloroform-methanol 95:5 (v/v) was used to resolve phenolic glycolipid-I (PGL-I) and a two-dimensional mobile phase of petroleum ether-ethyl acetate 98:2 (v/v) resolved three times for the first dimension, followed by petroleum ether-acetone 98:2 (v/v) in the second dimension was used to distinguish phthiocerol dimycocerosic acids (PDIM). TLC plates were imaged with a Typhoon 9400 scanner (G.E. Healthcare). The above assays were performed with and without the presence of the azasteroid, compound 1, that inhibits 3β-HSD activity (Thomas et al., 2011b). Compound 1 was dissolved in 100% DMSO, diluted to the appropriate concentration and incubated with *M. leprae* for 1 h before the addition of the [26-^14^C]cholesterol or [1-^14^C]palmitic acid. As a control, *M. tuberculosis* mc^2^6230 strain (3,2 x 10^8^ bacilli) was incubated with 100 µM compound 1 in Middlebrook 7H9 broth for 1 h at 37°C with agitation followed by addition of 1 µCi/mL [1-^14^C]palmitic acid and incubation at 37°C for 24 h.

17β-[N-(2,5-di-t-butylphenyl)carbamoyl]-6-azaandrost-4-en-3one (compound 1) was synthesized as previously described (Yang et al., 2019). The purity of the compound was assessed by NMR and HPLC to be >99% pure.

### Measurement of NAD^+^, NADP^+^ and Cytochrome C Reduction

The impact of 3β-HSD inhibition on NAD^+^, NADP^+^ and cytochrome C reduction was investigated using *M. leprae* whole cell lysate (WCL) (NR-19329) obtained from BEI Resources. Proteins were quantified in the lysates using Bradford protein assay (Biorad, Hercules, CA) (Bradford, 1976). The equivalent of 50 µg of protein from the WCL was used in the reaction performed in HBSS medium (ThermoFisher Scientific). WCL was incubated with either 200 µM NAD^+^ or 200 µM NADP^+^ (MilliporeSigma, St Louis, MO) in the presence of 200 µM cholesterol (MilliporeSigma) and treatment with or without 100 µM of compound 1. An assay of NAD^+^ and NADP^+^ reduction was performed every 30 sec for 20 min *via* absorbance measurement at 340 nm in an EON microplate spectrophotometer (BioTek Instruments, Inc. Winooski, VT). Similarly, the reduction of cytochrome C was measured at an absorbance of 550 nm.

### Schwann Cell Cholesterol Incorporation Assay

SCs were cultured in 24-well plates (Corning, Corning, NY) at a density of 5 x 10^4^ cells per well for flow cytometry and placed onto round 12 mm glass slips for confocal microscopy. Prior to *M. leprae* infection, the medium was replaced by fresh RPMI medium supplemented with 2% fetal calf serum. SCs were infected at a MOI of 50:1 with live *M. leprae* or stimulated with irradiated *M. leprae*, both fluorescently labeled with PKH26 (MilliporeSigma) according to manufacturer’s instructions. Infected or stimulated cultures were maintained at 33°C in 5% CO_2_ for 48 h. Cells were washed three times with PBS for 5 min and fresh RPMI medium without serum supplementation containing 0.1 mg/mL [BODIPY-Cho]-LDL (BODIPY 493/503, ThermoFisher) was added for labeling as described previously (Mattos et al., 2014). Flow cytometry was performed with the FACS Calibur Flow cytometer (BD, Holdrege, NE). For confocal microscopy the fixed cells were labeled with 2 µM DAPI (MilliporeSigma) for 1 min, washed with PBS and slides were prepared using Vectashield mounting medium (Vector Laboratories Inc, Burlingame, CA). Images were obtained with a LSM 510 Confocal Microscope (Zeiss, Oberkochen, Germany).

### Measurement of *M. leprae* Viability

For *M. leprae* intracellular viability, bacilli were incubated in RPMI medium supplemented with 0.05% BSA and 2% DMSO, with or without 100 µM compound 1, for 6 h at 30°C. *M. leprae* was collected by centrifugation at 16,000 x g, suspended in fresh RPMI medium and used to infect 1 x 10^5^ SCs at an MOI of 5:1. The infected SCs were incubated at 33°C for 24 h with 5% CO_2_. RNA was extracted using TRIzol reagent (ThermoFisher) as per manufacturer’s instructions. DNA was extracted after RNA isolation using 150 µL of chloroform and 100 µL of Tris-EDTA buffer (200 mM Tris 5 mM EDTA, pH 8) as described (Martinez et al., 2009). Isolated RNA samples were subjected to DNAse treatment using the Turbo DNA-free kit (ThermoFisher). cDNA was reverse transcribed from 500 ng of RNA using the GoScript kit (Promega) and diluted to 5 ng/µL. *M. leprae* viability was determined by qRT-PCR using *M. leprae* rRNA *16s* gene as molecular target as described (Martinez et al., 2009; De Toledo-Pinto et al., 2016).

To evaluate SC viability in the same experiments, 5 mg/mL of Thiazolyl Blue Tetrazolium Bromide (MTT) (MilliporeSigma) was added to each well 4 h prior to the end of the incubation period. Crystals were suspended in 100 µL 10% SDS and read at absorbance 590 nm using an EON microplate spectrophotometer (BioTek Instruments, Inc).

## Results

### *M. leprae* Increases the Uptake of LDL-Cholesterol by Infected Schwann Cells

**Figure 1:**
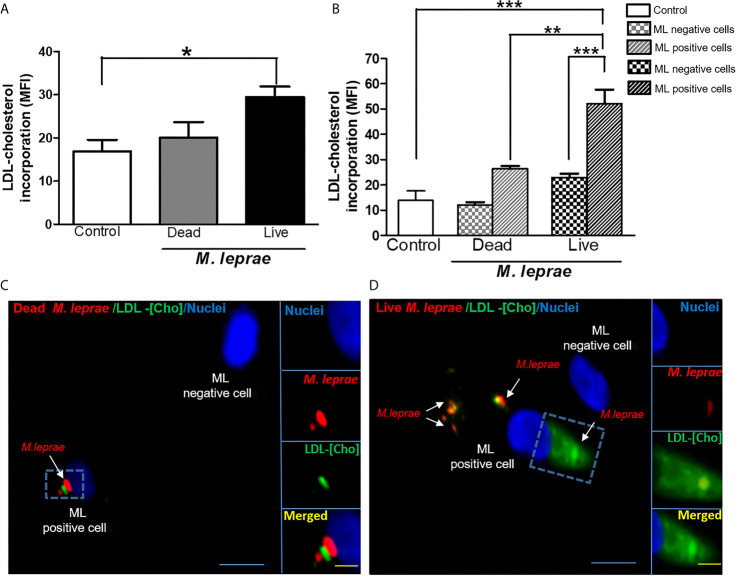
*M. leprae*-infected human SCs display increased uptake of LDL-cholesterol. ST88-14 human SC line was incubated with PKH26-labeled (red) *M. leprae* (ML) at a MOI of 50:1 for 48 h, followed by 2 h incubation with LDL-[Cho] (green). **(A, B)** LDL-[Cho] incorporation and bacterial association was measured simultaneously by flow cytometry and is expressed as medium fluorescence intensity (MFI). Results from three representative experiments are shown. Statistically significant differences (*p < 0.05 by Student’s t-test statistical analyses) (** p< 0.01 and ***p < 0.0001 - ANOVA test followed by Bonferroni as a post-test were performed and used for statistical analyses). **(C, D)** Confocal microscopy images showing LDL-cholesterol (green) incorporation in stimulated and infected cells. Dead and live *M. leprae* are labeled in red with PKH26 and nuclei are labeled in blue with DAPI. Arrows indicate colocalization of LDL-[Cho] with *M. leprae*. Scale bar, 5µm (blue) and 10µm (yellow).

Since the extracellular uptake of plasma lipoprotein-derived cholesterol (LDL-Cho) constitutes an important cellular lipid source, we analyzed whether *M. leprae* infection modulates LDL-Cho acquisition by SC. This pathway was investigated by monitoring the cellular internalization of fluorescent LDL. Cells were treated with dead or infected with live PKH-26-labeled *M. leprae*, pulse-labeled with LDL and lipid uptake was analyzed by flow cytometry. In comparison to the uninfected SC or SC treated with dead bacilli, LDL uptake increased in *M. leprae*-infected SC (MFI of 29.44 ± 2.403 in *M. leprae*-infected cultures and 20.05 ± 3.631 in dead *M. leprae*-treated (P=0.0252) (**Figure 1A**). Moreover, LDL uptake was significantly higher in SC bearing fluorescent-labeled bacteria in comparison to SC of the same culture that were devoid of bacteria or SC containing dead bacteria (**Figure 1B**). These data were confirmed by immunofluorescence images that clearly showed intense fluorescence of LDL-[Cho] (in green) in cells bearing live *M. leprae* (in red) when compared to uninfected cells and to cells treated with dead bacilli (**Figures 1C, D**). These images also revealed a close association between *M. leprae* and the just incorporated LDL-cholesterol in the host cell (**Figure 1D**).

### 3β-HSD Is the Enzyme in *M. leprae* Responsible for Converting Cholesterol to Cholestenone

**Figure 2:**
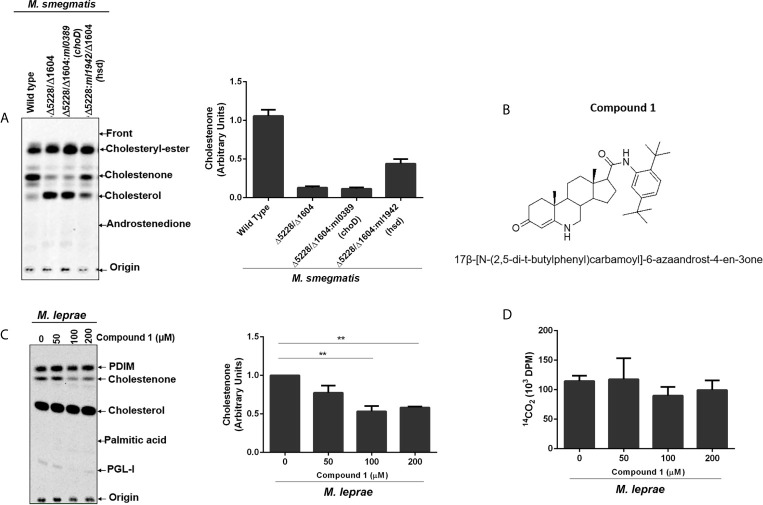
*M. leprae* 3β-HSD is the enzyme responsible for converting cholesterol into cholestenone. **(A)** Representative thin-layer chromatography (TLC) plate in silica G60 plates of Wild type (WT) *M. smegmatis*, hsd/choD double mutant (Δ5228/Δ1604) and double mutant complemented with either *M. leprae* choD (Δ5228/Δ1604:ml0389) or hsd (Δ5228/Δ1604:ml1942) were cultured in MSM-ty medium and incubated with [^14^C]cholesterol for 30 min to assess cholestenone production using petroleum ether-ethyl acetate 1:1 (v/v) as mobile phase. Corresponding densitometry for cholestenone is presented on the right (n=2). Reference standards: Cholesteryl-Ester; cholesterol; cholestenone; and Androstenedione. **(B)** Structure of compound 1, 17β-[N-(2,5-di-t-butylphenyl)carbamoyl]-6-azaandrost-4-en-3one. **(C, D)** *M. leprae* was incubated with increasing concentrations of compound 1 for 1 h in Middlebrook 7H9 broth supplemented with 2% DMSO at 30°C. 1µCi/mL of [26-^14^C]cholesterol and 1µCi/mL of [1-^14^C]palmitic acid were added to mycobacterium suspensions and incubated for additional 48 h at 33°C. **(C)** Lipid extracts obtained from mycobacterial cells by chloroform-methanol 2:1 (v/v) extraction were analyzed by TLC using chloroform-methanol 95:5 (v/v) as mobile phase. Radiolabeled lipids were observed in the PhosphorImager. Representative of 3 experiments and corresponding densitometry of cholestenone is depicted on the right. The cholestenone detection was determined using reference standards PDIM; cholesterol; cholestenone; palmitic acid and PGL-I. (**p < 0.01 - ANOVA test followed by Bonferroni as a post-test were performed and used for statistical analyses) (n=3). **(D)** *M. leprae* viability after compound 1 treatment was measured by radiorespirometry assay (n=3).

The capacity of *M. leprae* to incorporate cholesterol and convert it to cholestenone both *in vitro* and *in vivo* was previously demonstrated (Marques et al., 2015). To definitively establish the role of *M. leprae* 3β-HSD in the formation of cholestenone, *M. smegmatis* was used as a surrogate since genetic and functional studies are impaired by the inability to propagate *M. leprae in vitro*. Specifically, the *msmeg_5228* and *msmeg_1604* genes encoding the *M. smegmatis* 3β-HSD and ChoD homologues, respectively, were knocked out to generate the single and double mutant strains *(*Δ*msmeg_5228*, Δ*msmeg_1604* and Δ*msmeg_1604*/Δ*msmeg_5228*). The utilization of [4-^14^C] cholesterol by these strains revealed that, as expected, the production of cholestenone was a function of the *msmeg_5228* gene product (3β-HSD), but not *msmeg_1604* (ChoD) (**Supplementary Figure S1**). To confirm that 3β-HSD produced by *M. leprae* also converts cholesterol to cholestenone, the *ml1942* gene was expressed in the *M. smegmatis* Δ*msmeg*_*1604*/Δ*msmeg*_*5228* double mutant. As shown in **Figure 2A**, the production of cholestenone by the *M. smegmatis* double mutant was restored by the complementation with *ml1942* (*3β-HSD*). In contrast, cholestenone formation was not restored by complementation with *ml0389* (*choD)*. **Supplementary Figures S2A, B**, respectively, show that the *M. smegmatis* double mutant did not express the endogenous 3β-HSD and ChoD proteins, but was successfully complemented with the *ml1942* and *ml0389* genes, expressing the respective *M. leprae* 3β-HSD and ChoD homologues.

To further confirm the involvement of *M. leprae* 3β-HSD in cholesterol oxidation, we tested compound 1 (**Figure 2B**); an effective inhibitor of *M. tuberculosis* 3β-HSD (Thomas et al., 2011b). Since *M. leprae* 3β-HSD shares 85% similarity to the *M. tuberculosis* homologue, our expectation was that compound 1 would also inhibit the *M. leprae* enzyme. Live *M. leprae* was incubated in axenic medium with 1µCi/mL of [26-^14^C]cholesterol and 1µCi/mL of [1-^14^C]palmitic acid in the presence of increasing concentrations of compound 1. After 48 h, [1-^14^C] palmitic acid β-oxidation was measured by ^14^CO_2_ production (radiorespirometry) as an indicator of bacterial viability (Franzblau, 1988) and the impact on cholestenone production was analyzed by TLC. We observed approximately 50% inhibition in cholestenone production in bacilli treated with 100 µM or greater concentrations of compound 1 (**Figure 2C**), with a minimal effect on bacterial viability up to a concentration of 200 µM (**Figure 2D**). Compound 1 treatment also decreased cholestenone production by *M. smegmatis* Δ*msmeg*_*1604*/Δ*msmeg*_*5228*:*ml1942* (**Supplementary Figure S3**). Together these data confirm previous prediction that 3β-HSD is the sole enzyme responsible for cholestenone production in *M. leprae*. Moreover, our data revealed that, like in other mycobacteria, *ml0389* was incorrectly assigned to encode a cholesterol oxidase.

### 3β-HSD Inhibition Affects *M. leprae* Intracellular Survival

**Figure 3:**
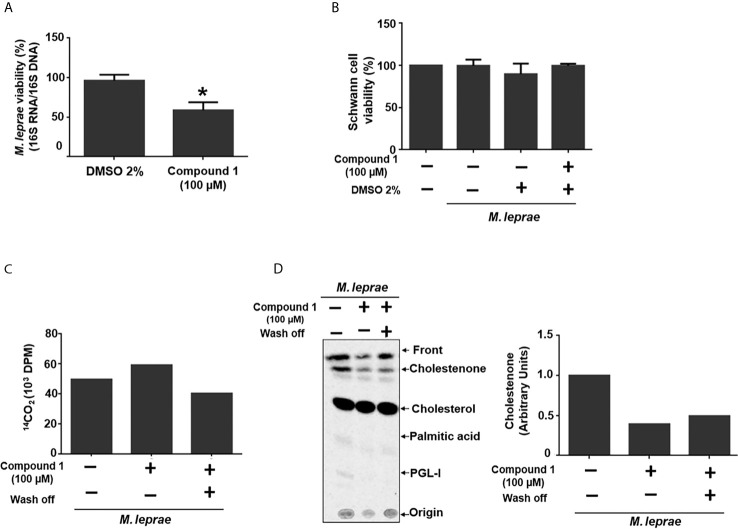
3β-HSD inhibition impacts *M. leprae* intracellular survival. **(A, B)** ST88-14 SCs were infected (MOI 5:1) for 24 h at 33°C with *M. leprae* pre-treated for 6 h with vehicle (2% DMSO) or 100 µM compound 1. **(A)** *M. leprae* intracellular viability was determined by qRT-PCR. (*p < 0.05 - by Mann-Whitney test)(n=5) **(B)** SC viability was measured by MTT assay (n=3). **(C, D)** *M. leprae* was incubated in axenic medium with 100 µM compound 1, 1µCi/mL of [26-^14^C]cholesterol and 1µCi/mL of [1-^14^C]palmitic acid for 6 h at 30°C followed by centrifugation and wash and removal of compound 1 (wash off, +) or replacement with fresh medium containing compound 1 (wash off, -), and incubation for 24 h at 33°C. **(C)** *M. leprae* viability was measured by radiorespirometry (n=1). **(D)** Radiolabeled lipids were resolved by TLC with chloroform-methanol 95:5 (v/v) as mobile phase and observed with a PhosphorImager. The graph indicates corresponding densitometry of cholestenone bands (n=1).

To investigate whether 3β-HSD activity is essential for *M. leprae* during infection, the impact of inhibition of the enzyme on bacterial intracellular survival was analyzed. Since mammalian cells also express proteins of the 3β-hydroxysteroid dehydrogenases superfamily, the bacteria were pretreated with compound 1, the inhibitor removed and SCs then infected in order to avoid potential effects on the host cell enzymes. This pretreatment of bacilli with the 3β-HSD inhibitor for 6 h accelerated bacterial killing by 30% after 24 h of infection (**Figure 3A**) and did not cause SC death (**Figure 3B**). The assay in axenic medium shown in **Figure 3C** confirms that bacterial viability is not affected by the drug after 24 h as already demonstrated in **Figure 2D** for an even longer time (48 h) of treatment. This assay also gives support to the result observed in **Figure 3A**. It shows that even removing compound 1 after 6 hours of incubation, inhibition of cholestenone production by *M. leprae* was sustained for the next 24 h (**Figure 3D**), reinforcing that the decrease in *M. leprae* intracellular viability observed in **Figure 3A** can be attributed to 3β-HSD inhibition. This data set strengthens the importance of cholesterol metabolism during *M. leprae* host cell interaction and brings attention to 3β-HSD as a new important element of this interaction.

### Cholesterol Oxidation by *M. leprae* 3β-HSD Generates NADH and NADPH

**Figure 4:**
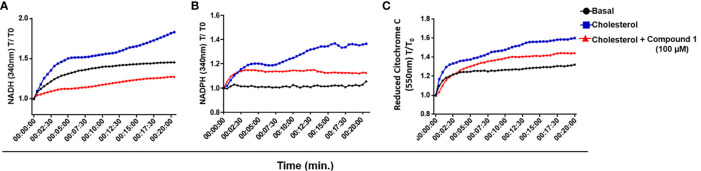
*M. leprae* 3β-HSD is a source of reductive power. **(A, B)** NAD^+^ and NADP ^+^ reduction to NADH and NADPH, respectively, by 3β-HSD activity was measured in a temporal kinetic curve every 30 s for 20 min at 340 nm. **(C)** Cytochrome C reduction was determined measuring the reduced form of Cytochrome C at 550 nm every 30 s for 20 min. In all conditions *M. leprae* WCL was incubated with 200 µM cholesterol, alone (blue) or in the presence of 100 µM compound 1 (red). A condition without cholesterol addition was also included as a control of basal levels (black). Representative of 3 independent experiments.

3β-HSD is a member of a family of enzymes that catalyzes an oxidation reaction with the consequent reduction of NAD^+^ to NADH or NADP^+^ to NADPH. *M. tuberculosis* 3β-HSD uses NAD^+^ instead of NADP^+^ (Yang et al., 2007) and *M. leprae* 3β-HSD has conserved the *M. tuberculosis* NAD^+^ binding motif homologue (Marques et al., 2015), suggesting that it also uses this cofactor. To test whether the catabolism of cholesterol could lead to the reduction of NAD^+^/NADP^+^ in *M. leprae*, a WCL of *M. leprae* was incubated with NAD^+^ or NADP^+^ in the presence or absence of cholesterol and reduction of the cofactors was measured spectrophotometrically by absorbance at 340 nm. As shown in **Figures 4A, B**, addition of cholesterol (blue curves) resulted in an increased generation of both NADH and NADPH, as compared to the levels observed in the absence of cholesterol (black curves). Moreover, blocking the 3β-HSD activity with compound 1 in the presence of cholesterol decreased both NADH and NADPH to levels close to or below the basal levels (red curves), reinforcing the fact that 3β-HSD is responsible for the increased reduction of these cofactors in the presence of cholesterol. Importantly, compound 1 was unable to affect the generation of NADPH when substrates for other dehydrogenases, such as citrate, acetate, or glyceraldehyde, were added to the cell extract (**Supplementary Figure S4**), confirming that compound 1 specifically inhibits the oxidation reaction catalyzed by 3β-HSD.

NADH represents a major electron donor feeding the respiratory chain; therefore, the NADH molecules generated by 3β-HSD activity could supply, at least in part, electrons to the oxidative respiratory chain contributing to *M. leprae* ATP synthesis. To check this possibility, the WCL of *M. leprae* was incubated in the presence or absence of cholesterol plus NAD^+^ and the reduction of cytochrome C was measured by absorbance at 550 nm (**Figure 4C**). An increased level of reduced cytochrome C was observed in the presence of cholesterol plus NAD^+^ (blue curve) as compared to baseline levels (black curve). Moreover, a partial decrease in this phenomenon was observed by blocking 3β-HSD activity with compound 1 (red curve). As expected, when NAD^+^ was replaced by NADP^+^ in identical assays no reduction of cytochrome C was observed (data not shown), emphasizing the specific participation of NAD^+^ in this process. These results suggest that cholesterol oxidation with the generation of electron donors can contribute to the *M. leprae* electron transport chain and, presumably, ATP generation.

### Cholesterol Oxidation by 3β-HSD Increases PGL-I and PDIM Synthesis

**Figure 5:**
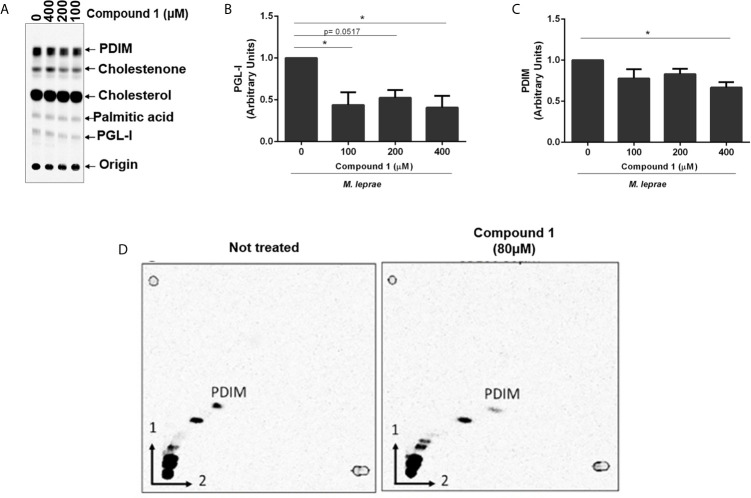
Reductive power generated by 3β-HSD supports PGL-I and PDIM synthesis. **(A)** TLC of lipid extracts from *M. leprae* treated with compound 1, representative of 4 experiments. TLC was resolved using chloroform-methanol 95:5 (v/v) as mobile phase. Standards: PDIM, cholesterol; cholestenone; palmitic acid and PGL-I. Corresponding densitometry of PGL-I **(B)** and PDIM **(C)** (*p < 0.05 - ANOVA test followed by Bonferroni post-test were performed for statistical analyses (n=4). **(D)** To confirm the apolar product labeled as PDIM in **(A)** was truly PDIM, 2-dimensional TLC analyses of the lipid extract from *M. leprae* treated or not with 80 µM compound 1 was performed.

Mycobacteria are enveloped by a notably complex cell wall structure, and many lipids are associated with the external leaflet. Primary examples of these lipids are the PDIM and PGLs (PGLs are phenolphthiocerol-based glycolipids that contain in its structure PDIM) (Marques et al., 1998). These components are polyketide-derived virulence factors, biosynthesis of which relies on electron donors, mainly NADPH. Since cholesterol oxidation generates reductive power, we investigated a potential link between 3β-HSD activity and bacterial lipid biosynthesis, focusing mainly on PDIM and PGL-I. The initial analysis of lipids produced in the presence of [26-^14^C]cholesterol and [1-^14^C]palmitic acid, demonstrated that incubation of *M. leprae* for 48 h with increasing concentrations of compound 1 resulted in decreased production of PGL-I and PDIM (**Figures 2C** and **5A**). A densitometric analysis of these TLC bands confirmed that compound 1 inhibited lipid biosynthesis in a dose-dependent manner (**Figures 5B, C**). The inhibition of PDIM biosynthesis, when 3β-HSD was blocked, was further confirmed by two-dimensional TLC analysis of lipid extracts of *M. leprae* incubated with or without compound 1 (**Figure 5D**). To evaluate whether compound 1 could act off-target (i.e. decrease lipid biosynthesis by mechanisms independent of 3β-HSD catalyzed cholesterol oxidation and consequent reduction of NADP^+^), *M. tuberculosis* was incubated with [1-^14^C]palmitic acid in the absence of cholesterol and treated or not with 100 µM compound 1, followed by measuring radiolabeled PDIM synthesis by TLC. PDIM synthesis was not affected by the presence of compound 1 (**Supplementary Figure S5**). Altogether, these results suggest that the reductive equivalents produced by cholesterol oxidation contribute to the biosynthesis of key *M. leprae* lipids.

## Discussion

A key aspect of *M. leprae* pathogenesis is its capacity to exploit host cell lipid metabolism, leading to the accumulation of cholesterol-enriched lipid droplets in the cytoplasm of infected cells (Virchow, 1863; Mattos et al., 2010; Mattos et al., 2011a; Mattos et al., 2014). In contrast to other mycobacteria that can fully degrade cholesterol, *M. leprae* has preserved only the capacity to oxidize cholesterol to cholestenone, the first step in cholesterol catabolism (Marques et al., 2015). In this study we explored the relevance of cholesterol oxidation in the context of *M. leprae*-SC interaction. Firstly, we showed that infected SCs display a higher capacity to import LDL, which colocalizes with intracellular bacilli, explaining, at least in part, *M. leprae-*induced cholesterol accumulation in the host cell. By using *M. smegmatis* mutant strains complemented with *M. leprae* genes, we confirmed that 3β-HSD (*ml1492)*, but not ChoD (*ml0389)*, is the enzyme responsible for cholesterol oxidation. Of note, treating *M. leprae* with compound 1, an inhibitor of 3β-HSD, decreased bacterial intracellular survival. Exploring the potential biochemical roles of this enzyme in *M. leprae* metabolism, we found that the reductive power generated by cholesterol oxidation can fuel the respiratory chain and potentially impact microbial ATP synthesis. In addition, 3β-HSD inhibition in turn inhibits the biosynthesis of the bacterial cell wall lipids PDIM and PGL-I. Altogether, our data suggest that the reductive power generated by cholesterol oxidation plays an essential role in *M. leprae* biology inside SCs, contributing to ATP and lipids synthesis.

The higher capacity of infected SCs to incorporate LDL-[Cho] suggests that *M. leprae* induces exogenous cholesterol uptake *via* upregulation of LDL receptors on the host cell surface. This same strategy was previously shown to be employed by *M. leprae* to induce cholesterol accumulation in macrophages (Mattos et al., 2014). Moreover, we observed an intimate physical association between the just incorporated LDL and intracellular *M. leprae*, in agreement with previous data showing the prompt recruitment and co-localization of LDs with internalized bacteria (Mattos et al., 2011a; Mattos et al., 2014). Altogether, these data reinforce the idea that host-derived lipids, such as cholesterol, promptly delivered to *M. leprae* containing phagosomes upon infection constitute a nutritional source essential for bacterial survival and persistence inside the host cell.

Our results demonstrated that only 3β-HSD of *M. leprae*, but not ChoD, was responsible for cholestenone production. These results were consistent with those obtained in the study of *M. tuberculosis* (Yang et al., 2011) and *M. smegmatis* (Ivashina et al., 2012). In fact, different studies have suggested an alternative role for ChoD during *M. tuberculosis* infection, connecting its importance to bacterial virulence with its capacity to down modulate the innate immune response through TLR-2 signaling pathway in infected macrophages (Brzostek et al., 2007; Klink et al., 2013). However, the unknown relevance of *M. leprae* retaining this single activity of cholesterol catabolism led us to investigate whether inhibition of 3β-HSD affected the viability of *M. leprae*. The treatment of *M. leprae* with the 3β-HSD inhibitor, compound 1 decreased cholestenone production; a result corroborated by compound 1 inhibition of cholestenone formation in the *M. smegmatis Δmsmeg_1604/Δmsmeg_5228* complemented with 3β-HSD of *M. leprae.* More importantly, treatment of *M. leprae* with compound 1 prior to infection of SCs resulted in decreased intracellular survival of the bacilli. These data provide strong support for the hypothesis that *M. leprae* utilizes cholesterol for intracellular survival (Mattos et al., 2014) and point to 3β-HSD as the linchpin for cholesterol dependent intracellular survival.

We hypothesized that the mechanism of *M. leprae* cholesterol dependent intracellular survival is rooted in the fact that 3β-HSD utilizes NAD^+^ as a cofactor. Indeed, the addition of cholesterol to WCL of *M. leprae* increased the reduction of NAD^+^, and this NAD^+^ reduction was inhibited by compound 1, reinforcing the specific involvement of 3β-HSD. Further evidence for the importance of NAD+ reduction by 3β-HSD was the observation that cholesterol plus NAD+ increased cytochrome C reduction when added to WCL of *M. leprae*, and blockage of 3β-HSD activity with compound 1 inhibited cytochrome C reduction. This suggests that the NADH molecules generated by 3β-HSD activity can fuel *M. leprae* electron transport chain contributing to ATP synthesis. Based on the genome sequencing data, it was originally suggested that *M. leprae* was unable to perform oxidative phosphorylation due to the lack of crucial genes of this pathway, especially genes that encode the NADH oxidase complex, which were found to be truncated (Cole et al., 2001). However, more recently it was reported that type II NAD dehydrogenase (NDH-2), encoded by the *ndh* gene (*rv1854c)* in *M. tuberculosis*, is involved in ATP synthesis (Rao et al., 2008). The *ndh* gene is present in the *M. leprae* genome *(ml2061*) and codes for a 466 amino acid protein sharing 90.2% similarity with the *M. tuberculosis* orthologue (Cole et al., 2001). Therefore, NADH produced by 3β-HSD activity could supply, at least in part, electrons to the oxidative respiratory chain.

Besides reduction of NAD^+^, *M. leprae* WCL incubated with cholesterol also generated NADPH, and this could be partially inhibited with compound 1. The *M. leprae* 3β-HSD shares high homology with the *M. tuberculosis* ortholog. However, the 3β-HSD of *M. tuberculosis* exclusively uses NAD^+^ as a cofactor and does not reduce NADP^+^. This cofactor-selectivity is suggested to be the result of an aspartate residue in position 45 of the primary amino acid sequence (Yang et al., 2007). Interestingly, *M. leprae* 3β-HSD also possesses an aspartate residue in the same position, suggesting that this enzyme would also use NAD^+^ exclusively as a cofactor (Marques et al., 2015). Thus, the inhibition of NADPH formation by compound 1 in the *M. leprae* extracts, could be an indirect consequence of NADH conversion to NADPH by other enzymes present in the bacterial extract. Prokaryotes present canonical and non-canonical reactions involved in the production and regeneration of NADPH. A NAD kinase (nicotinamide adenine dinucleotide kinase, NADK) is the known enzyme that generates NADP (H) by phosphorylating NAD (H) in almost all living organisms (Mori et al., 2005; Grose et al., 2006). As the major producer of NADP (H), NADK plays vital roles in maintaining the balance between NAD (H) and NADP (H) in NADP (H) -based cellular metabolic pathways (Kawai and Murata, 2008). The functionality of the NADK enzyme, encoded by the *ppnK* gene, has already been described in *M. tuberculosis* (Kawai et al., 2000) and a homologous gene, *ml1359*, is preserved in the *M. leprae* genome. There are also transhydrogenases that directly catalyze the reversible hydride transfer between NAD (H) and NADP (H). The *M. leprae* genome encodes two different isoforms of this enzyme, the energy-independent soluble transhydrogenase (STH) encoded by *ml1012c* and the energy-dependent, or proton-translocating, membrane-bound transhydrogenase (H + -TH) encoded by *ml2634c* or *ptnb*. Although *ml1012* is a pseudogene in *M. leprae*, the *ptnb* is predicted to be functional and could contribute to the formation of NADPH from NADH. In *E. coli* the H + -TH has been shown to provide about 40% of the total NADPH during growth on glucose (Sauer et al., 2004). Therefore, these enzymes could contribute to the pool of NADPH from the NADH generated by the action of 3β-HSD.

We observed that inhibition of 3β-HSD resulted in decreased synthesis of PGL-I and PDIM, two major constituents of *M. leprae* cell envelope, suggesting that the reductive power generated by 3β-HSD activity is being directed to lipid synthesis. This doesn’t seem to be an off-target effect of compound 1 treatment, since no alteration was observed in PDIM synthesis when *M. tuberculosis* was treated with the drug. This was an expected result as, in contrast to *M. leprae*, *M. tuberculosis* has a more versatile metabolic armament with the ability to generate reducing equivalents from various nutritional sources (Cole et al., 1998). Lipid anabolic pathways rely mainly on NADPH as a cofactor, although in the case of mycobacteria, the enzyme fatty acid synthase II (FAS II) presents one subunit that uses NADH (Marrakchi et al., 2000; Kruh et al., 2008). Despite the drastic reduction of the *M. leprae* genome, the collection of genes necessary for cell wall biosynthesis has been largely conserved; therefore, the reducing equivalents produced by the oxidation of cholesterol to cholestenone may be shifting from this pathway, contributing to the synthesis of PDIM and PGL-I. *M. leprae* has also preserved the gene coding for the transcription factor WhiB3, which may be involved in the effects of cholesterol oxidation on bacterial lipids biosynthesis. WhiB3 is part of a tight redox regulation system that senses variations in the redox balance and controls the anabolism of mycobacterial lipids such as polyacyltrehaloses (PAT), diacyltrehaloses (DAT), sulfolipids (SL-1) and PDIM (Saini et al., 2012; Mehta and Singh, 2019). WhiB3 activates lipid synthesis whenever there is an alteration in the redox state of the mycobacteria (Singh et al., 2009; You et al., 2019). So, it is reasonable to speculate that NADH/NADPH molecules generated upon *M. leprae* oxidation of cholesterol may result in WhiB3 activation and subsequent PGL-I and PDIM biosynthesis.

**Figure 6:**
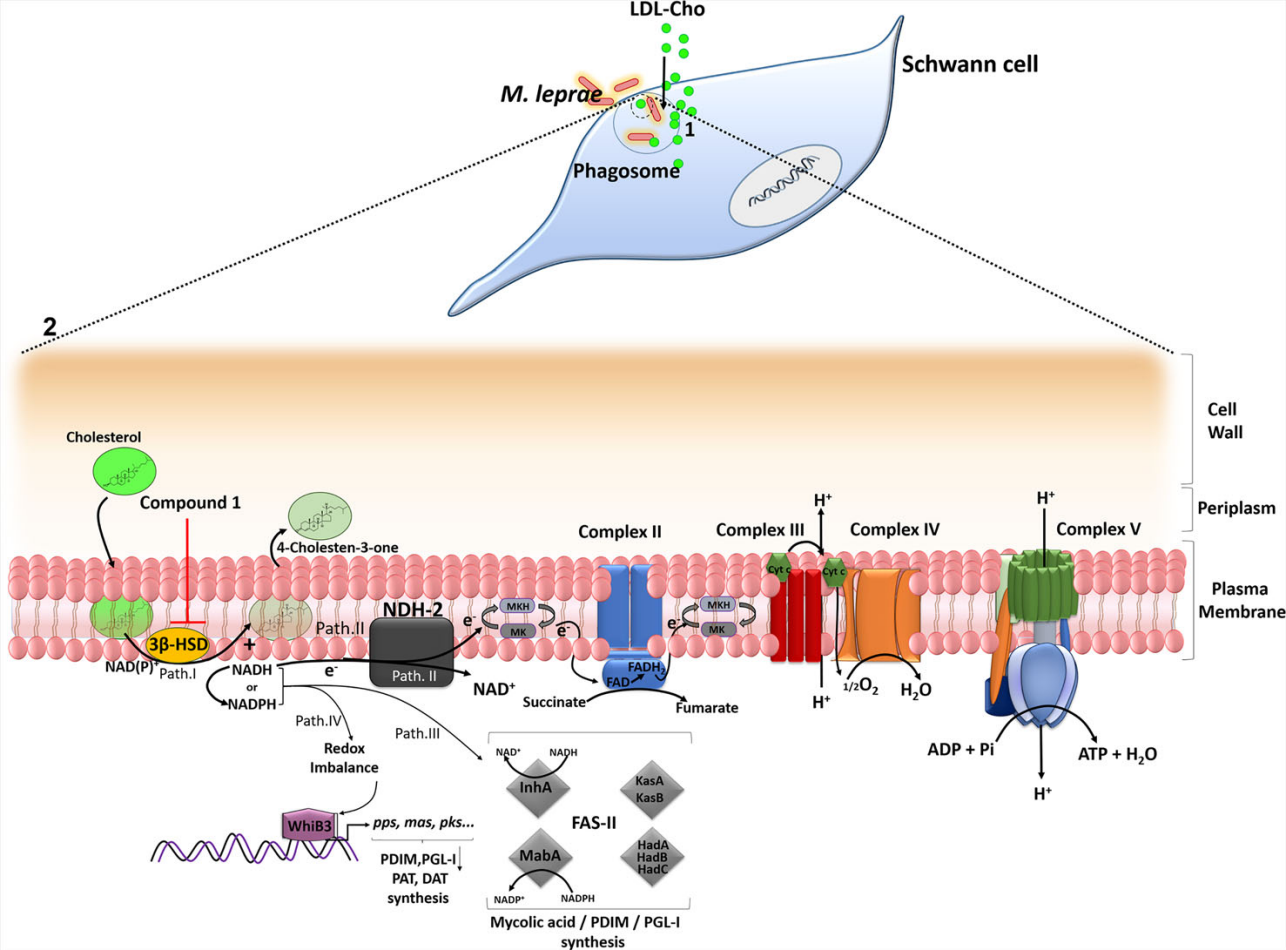
A model proposing a key role of cholesterol oxidation for *M. leprae* survival inside SCs. (1) After internalization, *M. leprae* induces LDL-cholesterol (LDL-Cho) uptake that is recruited to bacterium-containing phagosomes. (2) Magnification of *M. leprae* cell envelope. Path I - 3β -HSD oxidizes cholesterol to cholestenone generating NADH, which can be converted to NADPH. Path II - NADH derived from 3β-HSD feeds bacterial electron respiratory chain *via* type-II NADH dehydrogenase (NDH-2), which reduces menaquinone (MK) to menaquinol (MKH), a substrate of the succinate dehydrogenase (complex II). The proton motive force generated during electron transport chain will be used by the ATP synthase (complex V) to generate ATP. Path III – NADH/NADPH can be used by fatty acid synthase II (FASII) for the synthesis of PDIM and PGL-I. The FASII complex trans-2-enoyl-AcpM reductase (InhA) and β-ketoacyl-AcpM reductase (MabA) subunits use NADH and NADPH, respectively. Path IV - WhiB3, a probable transcriptional regulatory protein, senses variations in the redox balance through NADH and NADPH levels and activates the promoter region of polyketide biosynthetic genes inducing the synthesis of mycobacterial lipids such as polyacyltrehaloses (PAT), diacyltrehaloses (DAT), PDIM and PGL-I. Inhibition of 3β -HSD by compound **1** reduces the levels of NADH/NADPH affecting the metabolic pathways described and impacts *M. leprae* intracellular viability. The arrows indicate some of the destinations of NAD(P)H already described in the literature and propose routes fed by the reducing power generated from 3β–HSD.

In conclusion, our findings provide strong evidence that cholesterol oxidation *via* 3β-HSD is an important source of reductive power for the leprosy bacillus. A model can be proposed describing the key role of this enzyme in facilitating *M. leprae* persistence in the host cell (**Figure 6**). During reductive evolution, *M. leprae* has lost several oxidoreductases, oxidases and dehydrogenases and, although living in a similar intracellular environment, its capacity to degrade carbon and nitrogen substrates is considered limited when compared to *M. tuberculosis* (Cole et al., 2001; Borah et al., 2019). As a strategy to compensate this metabolic deficiency, *M. leprae* induces cholesterol accumulation and its recruitment to bacterium-containing phagosomes, which combined with the expression of 3β-HSD, turns this lipid into an easily available substrate for bacterial consumption and generation of reducing equivalents. The decrease in bacterial viability observed either by inhibiting cholesterol accumulation in the infected SCs (Mattos et al., 2011a) or by blocking 3β-HSD activity reinforces the hypothesis that cholesterol oxidation is an essential catabolic pathway for *M. leprae* pathogenesis, pointing to a novel drug target that may be used in combination with current multidrug regimens to diminish leprosy neuropathology.

## Data Availability Statement

The raw data supporting the conclusions of this article will be made available by the authors, without undue reservation.

## Author Contributions

TR and MM share first authorship and equally contributed with experiments and rational for the study. TR, MM, JB, MP, and MB-P were responsible for the study design and funding acquisition. TY and NS were responsible compound 1 production and supply. BV and CM were responsible to generate *M. smegmatis* knock out mutants. TR, MM, ZD, KH, CS, JA, and KM were responsible for investigation and performing experiments. GA was responsible for LDL-Cho supply and support with experiments using it. TR, MM, MB-P were responsible for results analysis. RL and PR provided ML for *in vitro* experiments. Project supervision was performed by MB-P, MP and JB. TR, MM, MB-P, PB, JB and MP were responsible for writing original manuscript draft. All authors contributed to the article and approved the submitted version.

## Funding

This project has been funded by National Institute of Allergy and Infectious diseases (NIAID) grant R01AI141526 and Conselho Nacional de Desenvolvimento Científico e Tecnológico (CNPq) grant 432434/2018-6.

## Conflict of Interest

The authors declare that the research was conducted in the absence of any commercial or financial relationships that could be construed as a potential conflict of interest.

## Publisher’s Note

All claims expressed in this article are solely those of the authors and do not necessarily represent those of their affiliated organizations, or those of the publisher, the editors and the reviewers. Any product that may be evaluated in this article, or claim that may be made by its manufacturer, is not guaranteed or endorsed by the publisher.

## Supplementary Material

Supplementary Figure 1 Oxidation of Cholesterol to cholestenone by *M. smegmatis* mutants. *M. smegmatis* Mc^2^155 wild type (WT) was mutated by recombineering. *msmeg_5228* (*hsd*) and *msmeg_1604* (*choD*) genes, coding, respectively, for the *M. smegmatis* 3β-HSD and ChoD homologues, were knocked out generating the single and double mutant strains Δ5228, Δ1604 and Δ1604/Δ5228. **(A, B)** *M. smegmatis* strains were incubated with [4-^14^C]cholesterol for 2h at 37°C followed by extensive washing with PBS-tyloxapol. Bacilli were extracted with chloroform-methanol (2:1), and the resulting lipids were analyzed by TLC using petroleum ether-ethyl acetate (1:1) as the running solvent. The graph indicates corresponding densitometry (n=2).

Supplementary Figure 2 Complementation of *M. smegmatis choD/hsd* double mutant with the *hsd* and *choD M. leprae* genes. *M. smegmatis* MC^2^ -155 wild type (WT-gray bar) and double knockout mutant (Δ5228/Δ1604- blue bar) were cultured in 457 minimal salt medium supplemented with 0,05 tyloxapol and 18 mM glycerol. Double knockout mutant complemented either *M. leprae ml0389* (*choD*) (Δ5228/1604:*ml0389* -green bar) or *M. leprae ml1942* (*hsd*) (Δ5228/1604:*ml1942* – red bar) were cultured in LB broth with 0,05% tyloxapol at 37°C and induced with 50 ng/mL Atc for 14 h. The cells were disrupted in 1mL of Trizol with 2 cycles in the TissueLyser and relative expression of **(A)** *M. smegmatis* genes: *msmeg_1604* (*choD*) and *msmeg_5228* (*hsd*) (n=2) or **(B)** *M. leprae* genes: *ml0389* and *ml1942* were determined by qRT-PCR (n=3).

Supplementary Figure 3 Compound 1 inhibit *M. leprae* 3β-HSD expressed in double mutant *M. smegmatis*. *M*. *smegmatis* Δ5228/1604:ml1942 was treated with compound 1 for 1h at 37°C with agitation, followed by further incubation in the presence of 1µCi/mL [4-^14^C]cholesterol for 1h. **(A)** Lipid extracts obtained from mycobacterial cells by chloroform-methanol 2:1 (v/v) extraction were analyzed by TLC using hexane-ethyl acetate 65:35 (v/v) as mobile phase. Radiolabeled lipids were observed in the PhosphorImager. **(B)** Representative of 2 experiments and corresponding densitometry of cholestenone.

Supplementary Figure 4 Compound 1 does not impact NADP reduction using substrates other than cholesterol. Acetate **(A)**, Glyceraldehyde **(B)** or Citrate **(C)**, well-known dehydrogenase substrates, were added to *M. leprae* whole cell sonicate and NADP^+^ reduction was determined measuring NADPH generation at 340 nm every 30 s for 20 min. Kinetics was performed in the absence of substrates (black), or with substrate alone (blue) or alongside treatment with 100 µM compound 1 (red).

Supplementary Figure 5 Absence of off-target effect of compound 1 on PDIM biosynthesis. *M. tuberculosis* (Mtb) was pre-incubated with 100 µM compound 1 for 1 h at 37°C followed by 1 µCi/mL [1-^14^C]palmitic acid addition and incubation for additional 24 h. Radiolabeled lipids were observed at the PhosphorImager after TLC with 2 runs of petroleum ether-ethyl acetate (98:2) as mobile phase. Representative of 2 experiments. Corresponding densitometry of PDIM is presented on the right.
