# Corrigendum: Reductive Power Generated by *Mycobacterium leprae* Through Cholesterol Oxidation Contributes to Lipid and ATP Synthesis

**DOI:** 10.3389/fcimb.2021.765326

**Published:** 2021-09-28

**Authors:** Thabatta L. S. A. Rosa, Maria Angela M. Marques, Zachary DeBoard, Kelly Hutchins, Carlos Adriano A. Silva, Christine R. Montague, Tianao Yuan, Julio J. Amaral, Georgia C. Atella, Patrícia S. Rosa, Katherine A. Mattos, Brian C. VanderVen, Ramanuj Lahiri, Nicole S. Sampson, Patrick J. Brennan, John T. Belisle, Maria Cristina V. Pessolani, Marcia Berrêdo-Pinho

**Affiliations:** ^1^ Laboratório de Microbiologia Celular, Instituto Oswaldo Cruz, Fundação Oswaldo Cruz, Rio de Janeiro, Brazil; ^2^ Department of Microbiology, Immunology and Pathology, Colorado State University, Fort Collins, CO, United States; ^3^ Department of Microbiology and Immunology, Cornell University, Ithaca, NY, United States; ^4^ Department of Chemistry, Stony Brook University, Stony Brook, NY, United States; ^5^ Laboratório de Química Biológica, Diretoria de Metrologia Aplicada às Ciências da Vida, Instituto Nacional de Metrologia, Qualidade e Tecnologia, Rio de Janeiro, Brazil; ^6^ Laboratório de Bioquímica de Lipídeos e Lipoproteínas, Instituto de Bioquímica Médica, Universidade Federal do Rio de Janeiro, Rio de Janeiro, Brazil; ^7^ Divisão de Pesquisa e Ensino, Instituto Lauro de Souza Lima, Bauru, Brazil; ^8^ Departmento de Controle de Qualidade, Instituto de Tecnologia em Imunobiológicos, Fundação Oswaldo Cruz, Rio de Janeiro, Brazil; ^9^ Department of Health and Human Services, Health Resources and Services Administration, Healthcare Systems Bureau, National Hansen’s Disease Programs, Baton Rouge, LA, United States

**Keywords:** *Mycobacterium leprae*, cholesterol, cholestenone, PGL-I, PDIM, 3β-HSD, reductive power, oxidation

## Error in Figure/Table

In the original article, there was a mistake in **Figure 4**:* M. leprae 3b-HSD is a source of reductive power* as published. Instead of original **Figure 4** image, the **Supplementary Figure 4** was duplicated and published as **Figure 4**, and the correct one is not on the published version of the article. The corrected **Figure 4** *: M. leprae 3b-HSD is a source of reductive power* appears below. The authors apologize for this error and state that this does not change the scientific conclusions of the article in any way. The original article has been updated.

**Figure 4:**
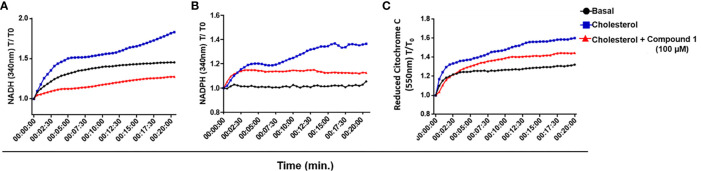
*M. leprae* 3β-HSD is a source of reductive power. **(A, B)** NAD+ and NADP + reduction to NADH and NADPH, respectively, by 3β-HSD activity was measured in a temporal kinetic curve every 30 s for 20 min at 340 nm. **(C)** Cytochrome C reduction was determined measuring the reduced form of Cytochrome C at 550 nm every 30 s for 20 min. In all conditions *M. leprae* WCL was incubated with 200 µM cholesterol, alone (blue) or in the presence of 100 µM compound 1 (red). A condition without cholesterol addition was also included as a control of basal levels (black). Representative of 3 independent experiments.

## Publisher’s Note

All claims expressed in this article are solely those of the authors and do not necessarily represent those of their affiliated organizations, or those of the publisher, the editors and the reviewers. Any product that may be evaluated in this article, or claim that may be made by its manufacturer, is not guaranteed or endorsed by the publisher.

