# Supplementary figures and images for: Reductive Power Generated by Mycobacterium leprae Through Cholesterol Oxidation Contributes to Lipid and ATP Synthesis
Source: Front Cell Infect Microbiol. 2021 Jul 28;11:709972. doi: 10.3389/fcimb.2021.709972 (PMC8355898; doi:10.3389/fcimb.2021.709972)

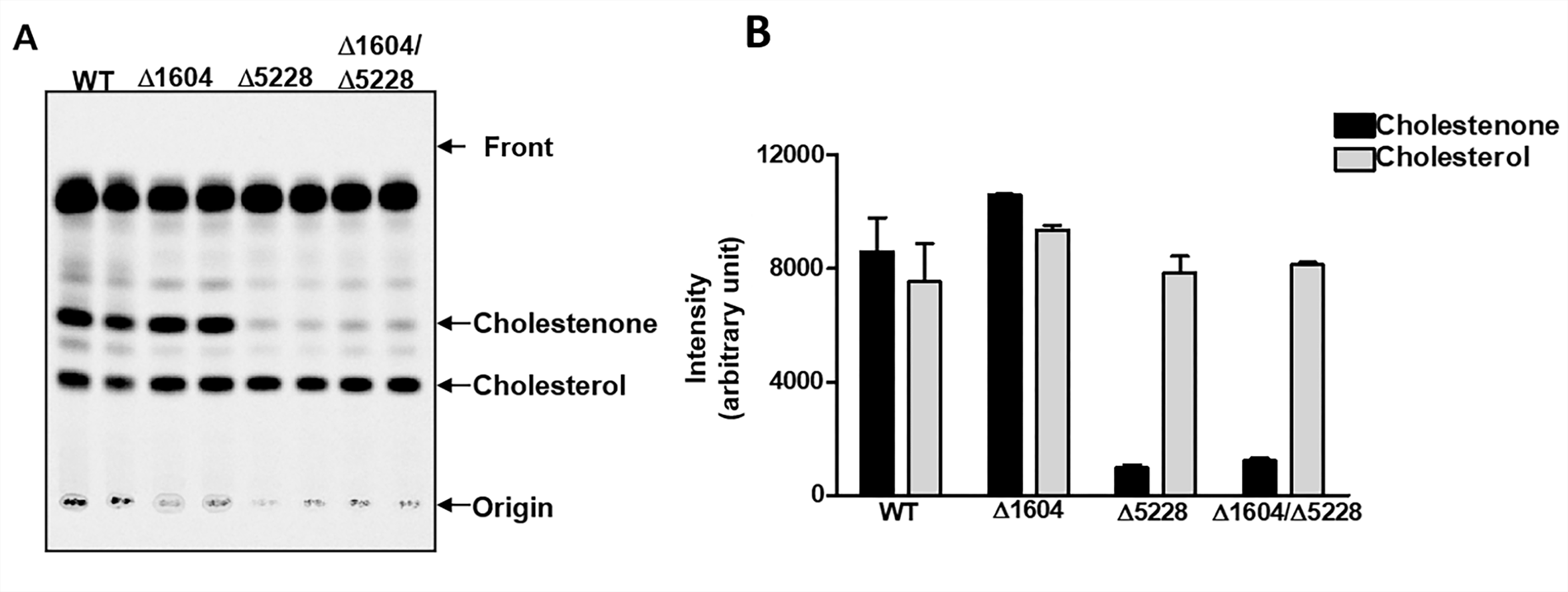

Supplement: Supplementary Figure 1 — Oxidation of Cholesterol to cholestenone by M. smegmatis mutants. M. smegmatis Mc2155 wild type (WT) was mutated by recombineering. msmeg_5228 (hsd) and msmeg_1604 (choD) genes, coding, respectively, for the M. smegmatis 3β-HSD and ChoD homologues, were knocked out generating the single and double mutant strains Δ5228, Δ1604 and Δ1604/Δ5228. (A, B) M. smegmatis strains were incubated with [4-14C]cholesterol for 2h at 37°C followed by extensive washing with PBS-tyloxapol. Bacilli were extracted with chloroform-methanol (2:1), and the resulting lipids were analyzed by TLC using petroleum ether-ethyl acetate (1:1) as the running solvent. The graph indicates corresponding densitometry (n=2). [file Image_1.tif]

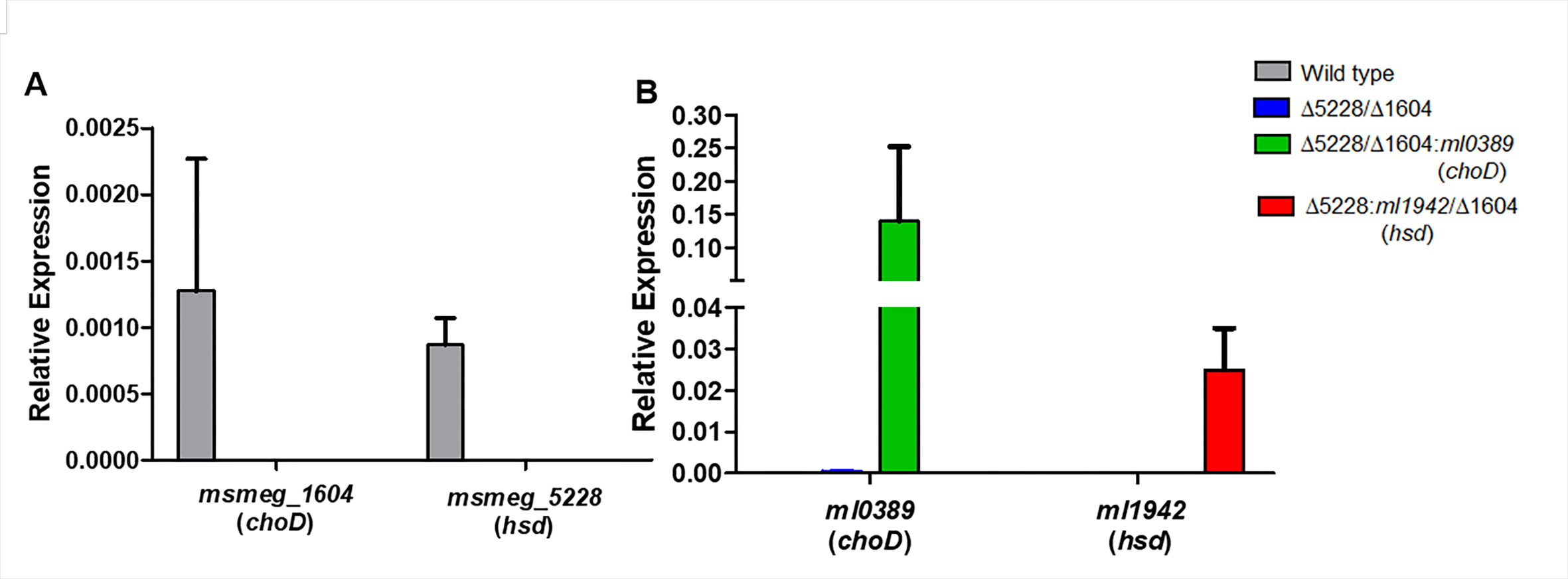

Supplement: Supplementary Figure 2 — Complementation of M. smegmatis choD/hsd double mutant with the hsd and choD M. leprae genes. M. smegmatis MC2 -155 wild type (WT-gray bar) and double knockout mutant (Δ5228/Δ1604- blue bar) were cultured in 457 minimal salt medium supplemented with 0,05 tyloxapol and 18 mM glycerol. Double knockout mutant complemented either M. leprae ml0389 (choD) (Δ5228/1604:ml0389 -green bar) or M. leprae ml1942 (hsd) (Δ5228/1604:ml1942 – red bar) were cultured in LB broth with 0,05% tyloxapol at 37°C and induced with 50 ng/mL Atc for 14 h. The cells were disrupted in 1mL of Trizol with 2 cycles in the TissueLyser and relative expression of (A) M. smegmatis genes: msmeg_1604 (choD) and msmeg_5228 (hsd) (n=2) or (B) M. leprae genes: ml0389 and ml1942 were determined by qRT-PCR (n=3). [file Image_2.tif]

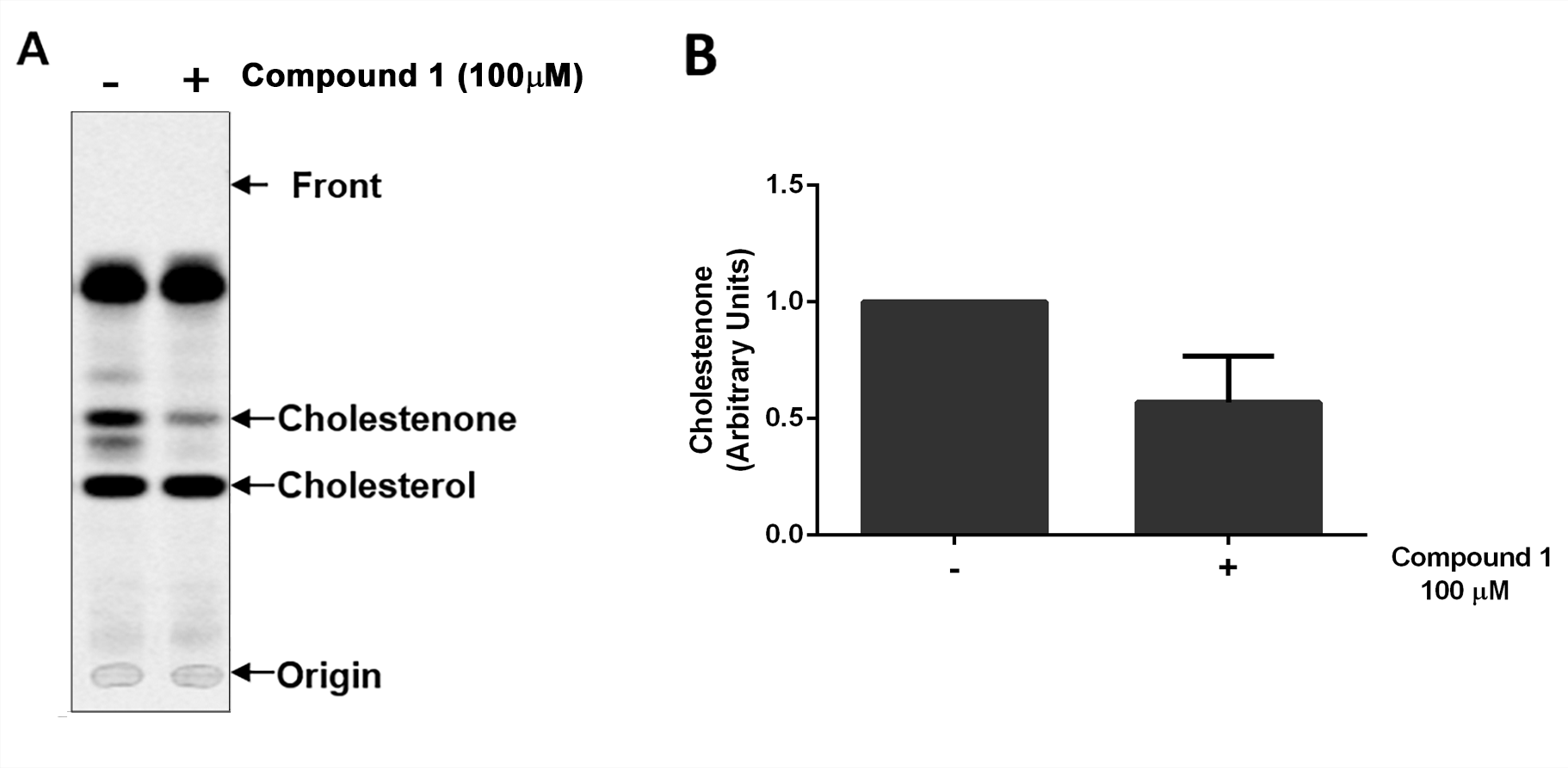

Supplement: Supplementary Figure 3 — Compound 1 inhibit M. leprae 3β-HSD expressed in double mutant M. smegmatis. M. smegmatis Δ5228/1604:ml1942 was treated with compound 1 for 1h at 37°C with agitation, followed by further incubation in the presence of 1µCi/mL [4-14C]cholesterol for 1h. (A) Lipid extracts obtained from mycobacterial cells by chloroform-methanol 2:1 (v/v) extraction were analyzed by TLC using hexane-ethyl acetate 65:35 (v/v) as mobile phase. Radiolabeled lipids were observed in the PhosphorImager. (B) Representative of 2 experiments and corresponding densitometry of cholestenone. [file Image_3.tif]

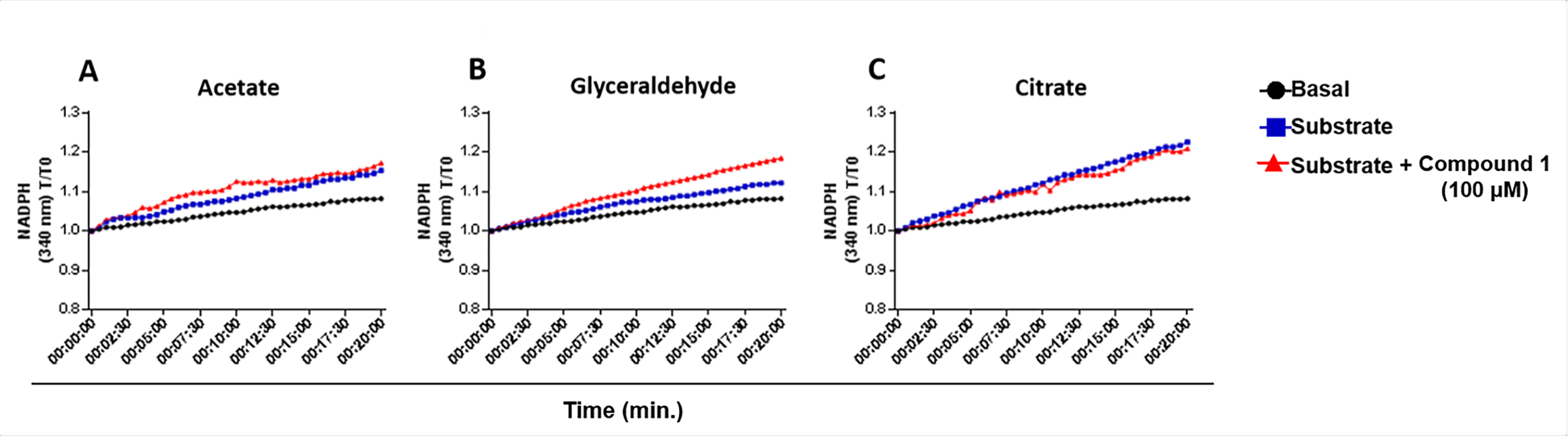

Supplement: Supplementary Figure 4 — Compound 1 does not impact NADP reduction using substrates other than cholesterol. Acetate (A), Glyceraldehyde (B) or Citrate (C), well-known dehydrogenase substrates, were added to M. leprae whole cell sonicate and NADP+ reduction was determined measuring NADPH generation at 340 nm every 30 s for 20 min. Kinetics was performed in the absence of substrates (black), or with substrate alone (blue) or alongside treatment with 100 µM compound 1 (red). [file Image_4.tif]

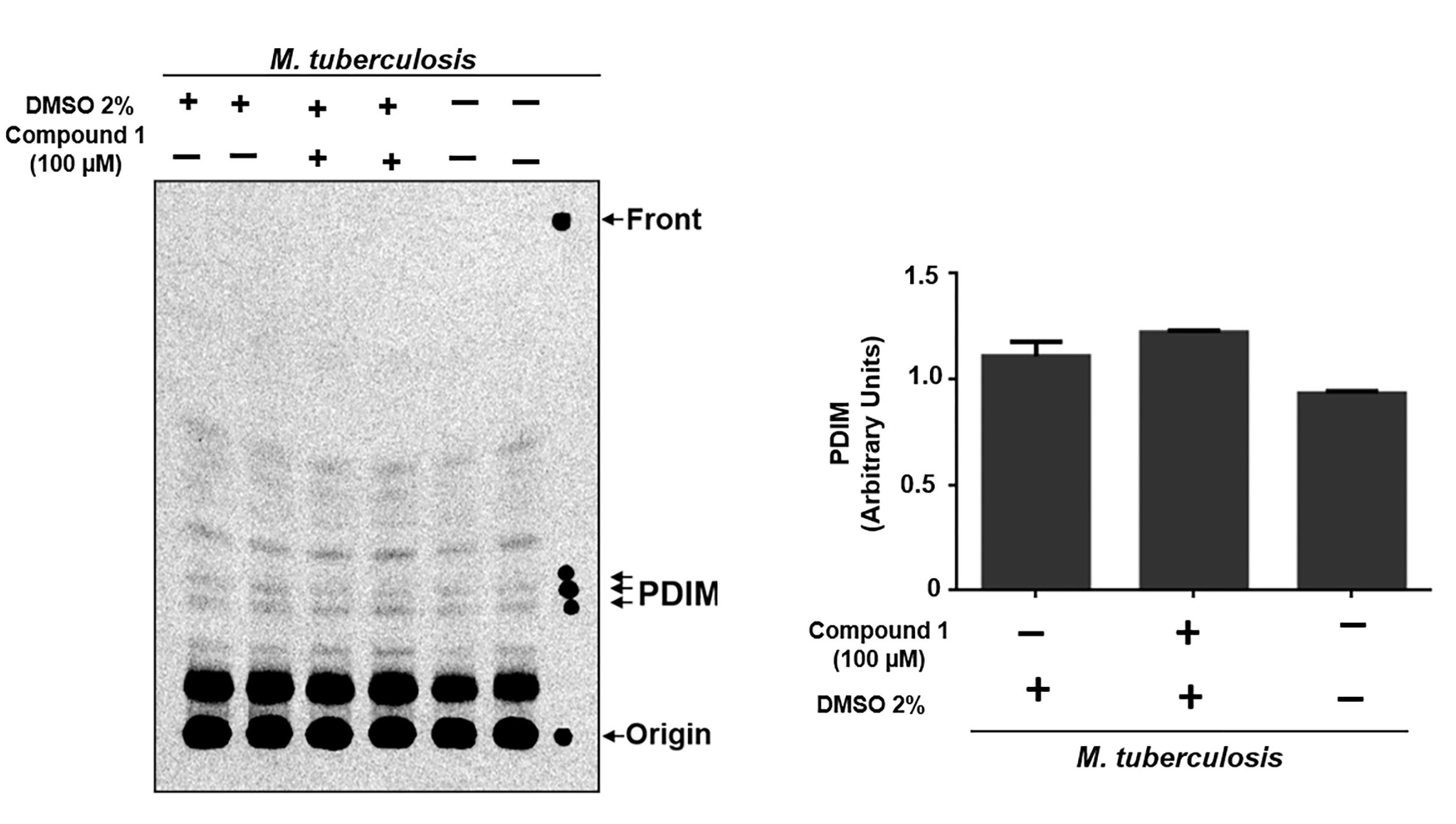

Supplement: Supplementary Figure 5 — Absence of off-target effect of compound 1 on PDIM biosynthesis. M. tuberculosis (Mtb) was pre-incubated with 100 µM compound 1 for 1 h at 37°C followed by 1 µCi/mL [1-14C]palmitic acid addition and incubation for additional 24 h. Radiolabeled lipids were observed at the PhosphorImager after TLC with 2 runs of petroleum ether-ethyl acetate (98:2) as mobile phase. Representative of 2 experiments. Corresponding densitometry of PDIM is presented on the right. [file Image_5.tif]
